# A Web Interface for Antibiotic Prescription Recommendations in Primary Care: User-Centered Design Approach

**DOI:** 10.2196/25741

**Published:** 2021-06-11

**Authors:** Ronni Madar, Adrien Ugon, Damir Ivanković, Rosy Tsopra

**Affiliations:** 1 Université Sorbonne Paris Nord Bobigny France; 2 ESIEE-Paris Noisy-le-Grand France; 3 Laboratoire d'Informatique de Paris 6 CNRS, Sorbonne Université Paris France; 4 Department of Public and Occupational Health Amsterdam Public Health Research Institute Amsterdam UMC, University of Amsterdam Amsterdam Netherlands; 5 Inserm, Université de Paris, Sorbonne Université, Centre de Recherche des Cordeliers, Information Sciences to support Personalized Medicine Paris France; 6 Inria Paris Paris France; 7 Department of Medical Informatics Hôpital Européen Georges-Pompidou Assistance Publique - Hôpitaux de Paris Paris France

**Keywords:** clinical decision support system, visualization, usability, clinical practice guidelines, antibiotic, primary care

## Abstract

**Background:**

Antibiotic misuse is a serious public health problem worldwide. National health authorities release clinical practice guidelines (CPGs) to guide general practitioners (GPs) in their choice of antibiotics. However, despite the large-scale dissemination of CPGs, GPs continue to prescribe antibiotics that are not recommended as first-line treatments. This nonadherence to recommendations may be due to GPs misunderstanding the CPGs. A web interface displaying antibiotic prescription recommendations and their justifications could help to improve the comprehensibility and readability of CPGs, thereby increasing the adoption of recommendations regarding antibiotic treatment.

**Objective:**

This study aims to design and evaluate a web interface for antibiotic prescription displaying both the recommended antibiotics and their justifications in the form of antibiotic properties.

**Methods:**

A web interface was designed according to the same principles as e-commerce interfaces and was assessed by 117 GPs. These GPs were asked to answer 17 questions relating to the usefulness, user-friendliness, and comprehensibility and readability of the interface, and their satisfaction with it. Responses were recorded on a 4-point Likert scale (ranging from “absolutely disagree” to “absolutely agree”). At the end of the evaluation, the GPs were allowed to provide optional, additional free comments.

**Results:**

The antibiotic prescription web interface consists of three main sections: a clinical summary section, a filter section, and a recommended antibiotics section. The majority of GPs appreciated the clinical summary (90/117, 76.9%) and filter (98/117, 83.8%) sections, whereas 48.7% (57/117) of them reported difficulty reading some of the icons in the recommended antibiotics section. Overall, 82.9% (97/117) of GPs found the display of drug properties useful, and 65.8% (77/117) reported that the web interface improved their understanding of CPG recommendations.

**Conclusions:**

The web interface displaying antibiotic recommendations and their properties can help doctors understand the rationale underlying CPG recommendations regarding antibiotic treatment, but further improvements are required before its implementation into a clinical decision support system.

## Introduction

Antibiotic misuse is a serious public health problem worldwide [1,2]. It exposes patients to the risk of adverse effects and complications, including death [3,4], as well as bacterial resistance [5]. Most antibiotic prescriptions are made in primary care settings. In this context, the choice of antibiotic is usually empiric (ie, without identification of the causative bacterium) and depends on various microbiological, epidemiological, pharmacological, patient condition–, and general practitioner (GP)-related factors [6].

National health authorities release clinical practice guidelines (CPGs) to guide GPs in their choice of antibiotics. CPGs contain evidence-based recommendations from a group of experts based on scientific publications. However, despite the large-scale diffusion of CPGs, GPs continue to prescribe antibiotics that are not recommended for first-line treatment (eg, broad-spectrum antibiotics) [7,8]. This noncompliance with recommendations may be due to the GPs misunderstanding the CPGs or their lack of confidence in these guidelines [9,10]. Indeed, it has been shown that GPs are suspicious of the content of CPGs; they believe that the recommendations are driven by economic issues rather than a desire to improve patient care and that there is a lack of evidence to support the recommendations [11]. They also find the guidelines unclear, ambiguous, incomplete, complex, and unusable in clinical practice [11].

An antibiotic prescription web interface displaying not only recommendations of antibiotics but also their justifications could help improve the GPs’ comprehensibility and readability of CPGs, thereby increasing the adoption of recommendations regarding antibiotic treatment. The justifications for antibiotic recommendations can be found within CPG documents, but they are often *lost* or *hidden* in large amounts of text [12,13]. A qualitative analysis of CPGs [12,13] for antibiotic treatment showed that these justifications were based on antibiotic properties [14,15]. For example, fosfomycin trometamol is recommended for the treatment of uncomplicated cystitis because of the following properties: short-duration protocol, few side effects, and little collateral damage. The display of antibiotic properties in an easily accessible and understandable manner could therefore help improve the comprehensibility and readability of CPGs for GPs, thereby increasing the chances of them successfully adopting those recommendations.

It is not easy to display the recommended antibiotics and their properties in a readily usable interface [16,17]. In the context of antibiotic treatment, several kinds of interface have been used, including textual formats [18,19], tables [19], diagrams [20], hypertextual links [21], and tick boxes [18]. Outside the domain of medicine, e-commerce interfaces (such as those used by e-commerce organizations like Booking, eBay, and Amazon) are widespread and make it possible for consumers to compare particular parameters between products. However, surprisingly, interfaces of this type are rarely used in the medical domain. Such e-commerce interfaces could be an effective way of displaying both antibiotics and their properties.

In this study, we aim to (1) design a web interface for antibiotic prescription presenting recommendations and justifications, in the form of an e-commerce interface displaying antibiotics and their properties, and (2) evaluate the readability and utility of this interface for improving the GPs’ comprehension of CPGs.

## Methods

### Interface Design

The elements involved in medical decision-making for antibiotic prescription have been previously identified based on analyses of clinical guidelines [14] and clinical expertise [6].

**Figure 1:**
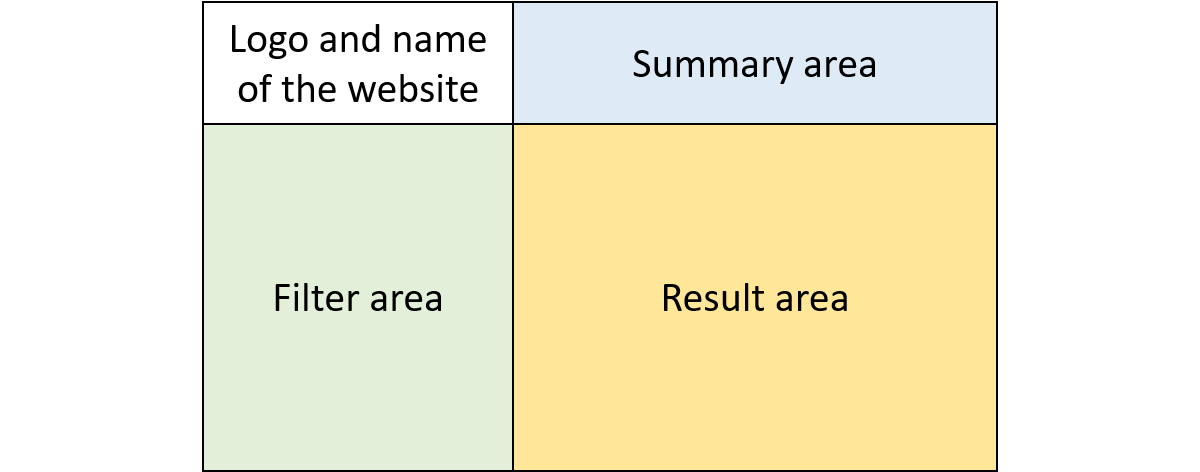
Basic wireframe of e-commerce interfaces compartmentalized into sections.

The best way to organize these elements into the proposed web interface was determined by reviewing and analyzing the content of the following:

1. Health care interfaces: However, no interface with a design similar to that of an e-commerce interface was retrieved.
2. Several well-known e-commerce websites for booking trips or hotels and web-based stores: A panel of websites were reviewed and the interface of four selected e-commerce websites (ie, Booking, Amazon, Tripadvisor, and LeBonCoin) were analyzed in greater detail. This analysis revealed that these interfaces contained three common main sections: a summary section, a result section, and a filter section (Figure 1). The same partitioned sections were then used to organize the elements involved in medical decision-making and to design the web interface.

The *summary* section of e-commerce interfaces usually displays the search criteria entered by the user when searching for a product (eg, “hotels in Paris from 21/12/2020 to 26/12/2020”). In our web interface, the elements “patient profiles” and “diseases” (eg, adult pharyngitis) were considered as search criteria.

The *filter* section of e-commerce interfaces usually displays optional parameters that users can click to filter the products selected in response to the initial search criteria (eg, hotel price). In our web interface, elements relating to certain generic patient conditions that are taken into account during drug prescription (eg, renal failure) were considered as optional parameters.

Finally, the *result* section of e-commerce interfaces usually displays the products recommended after both the search criteria and the optional parameters entered by the user have been taken into account. The characteristics of the products are usually also displayed in the form of icons to facilitate product comparison (eg, an icon for a swimming pool). In the web interface, the elements “antibiotic” and “antibiotic properties” were considered as products and characteristics, respectively. The antibiotics recommended in CPGs and the properties used by CPG experts to make those recommendations would then be displayed. A previous study identified seven preference properties (eg, “convenient protocol”) that are currently used by experts for making recommendations [14]. Antibiotics satisfying these properties are preferred over others, depending on the clinical situation (eg, in women with uncomplicated cystitis, fosfomycin trometamol is preferred over other antibiotics because it has the property “convenient protocol”). For each preference property, two junior doctors reviewed the icon web sites and designed personalized icons using Inkscape. The seven preference properties, as described by CPG experts, are shown along with the representative icons in Table 1.

**Table 1:** Icons used for displaying antibiotic properties.

Preference property	Definition^a^	Icon description	Icon
Convenient protocol	If the antibiotic is prescribed orally and for less than Z days^b^	Symbolized by a capsule placed on a hand	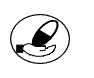
Absence of serious and frequent side effects	If there is no risk of serious side effects and the frequency of side effects is sufficiently low to allow prescription	Symbolized by a face with skin rash with an interdictory stroke	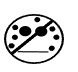
Nonprecious class	If the antibiotic does not belong to a class of drugs that must be preserved for more serious infections	Symbolized by a diamond with an interdictory stroke	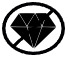
Narrow antibacterial spectrum	If the antibiotic is described as having a narrow antibacterial spectrum	Symbolized by mass spectrum graph	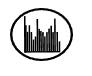
Low level of ecological adverse effects	If the antibiotic is described as having a low risk of promoting bacterial resistance	Symbolized by a recycling icon	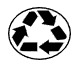
High level of efficacy	If the antibiotic is described as very effective (eg, high clinical cure percentage)	Symbolized by brachial biceps with a boxing glove	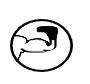
Acceptable taste	If the antibiotic is described as having an acceptable taste for oral administration	Symbolized by a face with tongue emoji	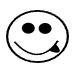

^a^Expert definitions within clinical practice guidelines. Source: [14].

^b^Z: the period depends on the clinical situation.

### Interface Assessment

The utility of the interface for improving GPs’ comprehension of CPGs and their readability was evaluated using a questionnaire tailored to the study.

#### GP Recruitment

GPs were contacted via emails sent to medical networks in the Île-de-France region (including Paris) and by word of mouth, between March 25 and April 25, 2018. A reminder was sent 15 days after the first contact was made. For inclusion in the study, GPs had to be in training or practicing in primary care. The evaluation was voluntary and anonymous.

#### Study Design

A web-based evaluation was carried in three steps. First, the participating GPs were asked to answer five sociodemographic questions. Their responses were rendered anonymous. Next, the GPs were asked to use the web interface for the use case “young adult woman with uncomplicated pyelonephritis.” The information displayed on the web interface was derived from a knowledge base describing 11 infectious diseases, 50 antibiotics, and 21 patient profiles, constructed based on French CPGs and clinical expertise (a description of this knowledge base is available in the literature [14]). Then, the GPs were asked to answer 17 questions relating to usefulness (3 questions), user-friendliness (3 questions), satisfaction (3 questions) and comprehension (8 questions). The responses were recorded on a 4-point Likert scale (ie, “absolutely disagree,” “tend to disagree,” “tend to agree,” and “absolutely agree”). At the end of the evaluation, GPs were provided with an opportunity to write optional free comments.

## Results

### Web Interface for Antibiotic Prescription

**Figure 2:**
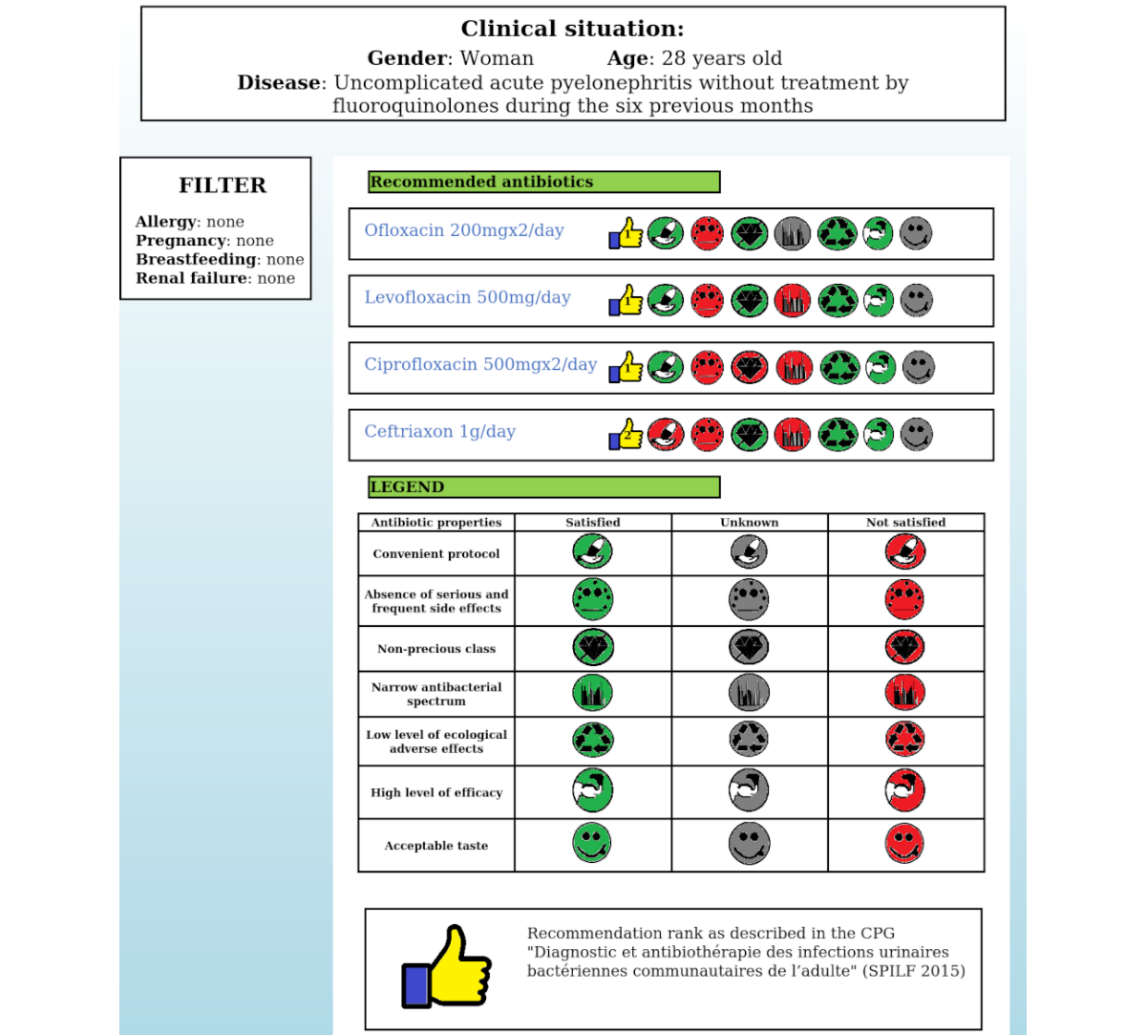
Web interface for antibiotic prescription displaying recommended drugs and their properties.

The web interface for antibiotic prescription was divided into three main sections, as in most e-commerce websites (Figure 2):

1. The *clinical summary* section, located at the top of the interface, displays a short summary of the clinical situation defined by both the disease and the profile of the patient (eg, sex, age).
2. The *filter* section, located at the top left of the interface, can be used to filter the recommended drugs according to patient-specific conditions such as drug allergies, renal failure, pregnancy, and/or breastfeeding.
3. The *recommended antibiotics* section, located in the center of the interface, displays the recommended drugs, with justifications in the form of drug properties. These preference properties are represented by seven icons. Each icon is shown in a different color, as follows: green, if the recommended antibiotic satisfies the property; red, if the recommended antibiotic does not satisfy the property; and grey, if no information is available. For each recommended drug, the recommendation rank is displayed with a numbered thumbs-up icon, as per the CPGs.
4. The *legend* located at the bottom of the interface highlights the icons used in the recommended antibiotics section and displays hypertextual links to the original CPGs.

### Evaluation of the Antibiotic Prescription Web Interface

#### Characteristics of Participating GPs

Given the opportunistic nature of the recruitment method used, it is difficult to estimate accurately how many GPs received the invitation email. The number of GPs contacted was estimated to range between 850 and 880.

In total, 117 GPs working within distinct GP surgeries accepted the invitation and assessed the web interface. More than half (73/117, 62.4%) were female. More than three-fourths (104/117, 89.0%) were under 40 years of age and had been in practice for less than 10 years. More than half (77/117, 65.8%) were working in private practice (Table 2).

**Table 2:** Sociodemographic characteristics of the general practitioners (GPs) (N=117).

Characteristic			Value, n (%)
**Sex**			
	Female	73 (62.4)	
	Male	44 (37.6)	
**Age (years)**			
	20-30	42 (36.0)	
	30-40	62 (53.0)	
	40-50	7 (6.0)	
	50-60	3 (2.5)	
	>60	3 (2.5)	
**Professional status**			
	GP with an MD thesis	74 (63.3)	
	GP without an MD thesis	39 (33.3)	
	GP in training	4 (3.4)	
**Time in practice (years)**			
	<5	81 (69.2)	
	5-10	20 (17.1)	
	10-20	10 (8.5)	
	>20	6 (5.2)	
**Practice type**			
	Private	77 (65.8)	
	Salaried	15 (12.8)	
	Mixed	25 (21.4)	

#### Satisfaction, Usefulness, Comprehensibility, and Readability of the Web Interface

**Figure 3:**
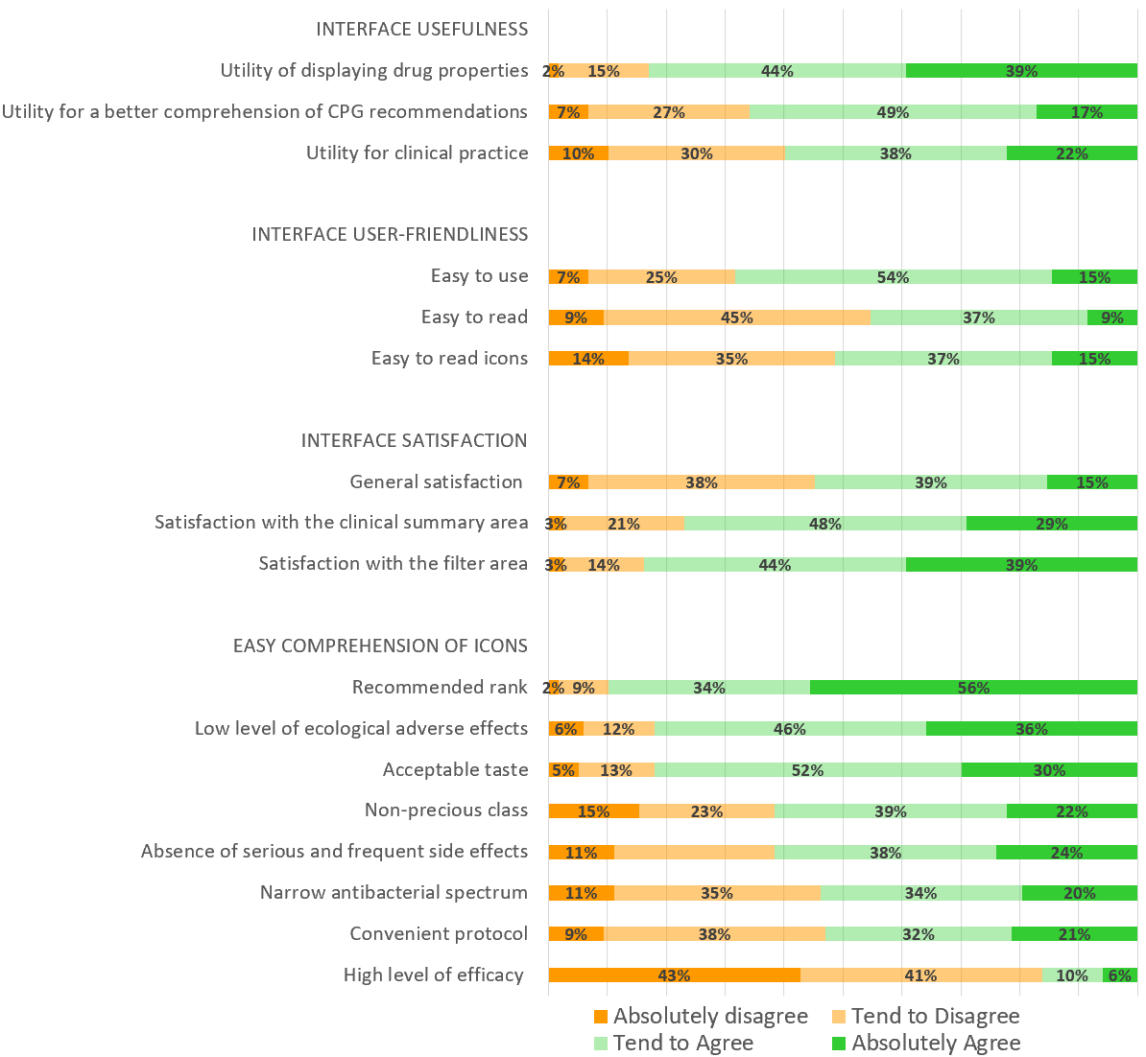
Satisfaction, usefulness, readability, and comprehensibility of the web interface according to general practitioners. In the questionnaire, each item was turned into an affirmative sentence to be graded on a 4-point Likert scale ranging from “absolutely disagree” to “absolutely agree” (eg, the item “Utility of displaying drug properties” was converted to the statement “I found the display of drug properties useful” and this affirmation was then graded).

Overall, 82.9% (97/117) of GPs found the display of drug properties useful (95% CI 76-90); 65.8% (77/117) reported that the interface improved their understanding of CPG recommendations (95% CI 57-74), and 59.8% (70/117) considered the interface useful for clinical practice (95% CI 51-69), as shown in Figure 3.

Only 54.7% (64/117) of all GPs were satisfied with the global interface (95% CI 46-64), probably because of dissatisfaction with some of the icons. Indeed, the majority of GPs appreciated the clinical summary (90/117, 76.9%) and filter (98/117, 83.8%) sections, whereas 48.7% (57/117) of them reported difficulties understanding some of the icons (Figure 3).

Five of the eight icons were considered sufficiently readable. The icons for recommended rank, ecological adverse effects, and taste were considered easily understandable by more than 80% of GPs; both the precious class and the side effects icons were considered comprehensible by more than 60% of all GPs. By contrast, three icons were not sufficiently readable; icons for antibacterial spectrum and convenient protocol were considered poorly understandable by nearly half the GPs and that for efficacy level was considered poorly understandable by about 84% of GPs (Figure 3).

#### Areas for Improvement

Some GPs said that they found the web interface interesting but that it could take time for them to get familiar with it. They suggested several areas for improvement (Table 3).

For the clinical summary section, GPs suggested displaying more details about the probable causal microbes, patient history, treatment, and signs of gravity. For the filter section, they suggested adding filters for hepatic conditions and galenic forms. Regarding the recommended antibiotics section, the GPs thought it included too much information and needed to be simplified to improve readability. For example, some GPs said that only the most important properties should be displayed and that other properties should be hidden and accessible only on request (eg, nonprecious class, efficacy level, and taste). Surprisingly, some GPs reported that some properties were not important for the choice of antibiotic, whereas these properties were used by CPG experts in the formulation of recommendations (eg, ecological adverse effects, activity spectrum, antibiotic taste). Other GPs also had doubts about the nonprecious class property because they did not understand what this meant. Conversely, other GPs suggested including additional properties such as drug cost, drug-drug interactions, and contraindications. Some others said that some of the icons were not sufficiently intuitive and required revision. They suggested, for example, replacing the efficacy level by a graduated scale, speedometer, or weapon symbol. Finally, some GPs said that they thought the web interface could be useful for shared decision-making with patients.

**Table 3:** Areas for improvement extracted by analysis of free comments by general practitioners (GPs) (N=117).

Interface section and suggested improvement		GP, n (%)
**Clinical summary**		
	Add more information about patient history and current treatment	9 (7.7)
	Add more information about clinical signs of gravity	1 (0.9)
	Add details about the suspected causal microbes	1 (0.9)
**Filter**		
	Add hepatic condition filter	1 (0.9)
	Add galenic form preference (eg, syrup, pill)	2 (1.7)
**Recommended antibiotics**		
	Improve readability	31 (26.5)
	Add some properties (eg, duration, cost)	8 (6.8)
	Specify important patient contraindications for each antibiotic	2 (1.7)
	Specify drug-drug interactions	3 (2.6)

## Discussion

### Principal Findings

An interface for antibiotic prescription displaying the recommendations and their justifications was designed and assessed. The web interface was divided into three main sections: a clinical summary section, a filter section, and a recommended antibiotics section enlisting recommended antibiotics and their properties displayed as icons. Overall, 82.9% of participating GPs found the display of drug properties useful, and 65.8% of them reported that the interface helped to improve their understanding of CPG recommendations. Nevertheless, the interface requires further improvement before its implementation in a clinical decision support system (CDSS).

### Limitations

#### Interface Design

##### Icon Design

Despite 82.9% of the GPs reporting that they found the display of antibiotic properties useful, 48.7% reported difficulty in reading the corresponding icons. Starren et al [22] described five kinds of presentation for displaying medical data (ie, table, list, graph, generated text, and icon). We believed that icons were the most suitable presentation for displaying the antibiotic properties because they are small pictorial symbols that are particularly well adapted for displaying qualitative data within computer interfaces [22]. Furthermore, they are already widely used for displaying medical data such as for medication administration or patient events [22]. Iconic languages [23-25] have even been developed in the medical domain, such as the Visualisation des Connaissances Médicales (Visualization of Medical Knowledge [VCM]) language [25,26], which is used to represent signs, diseases, physiological states, life habits, and drugs. However, as there were no existing icons for representing antibiotic properties, the icons for this interface were designed by ourselves. Designing unambiguous icons for displaying precise concepts, such as “low level of ecological adverse effects” or “narrow antibacterial spectrum” was not an easy task, and it can take time for the GPs to become familiar with these icons. In our study, GPs discovered the icons for the first time during the evaluation period, with no prior tutorial or training, potentially accounting for their lack of satisfaction with the existing icons. The next step will be improving the design of some icons in accordance with the suggestions made by the GPs participating in this study (eg, “efficacy level” could be represented by a graduated scale). A tutorial and interactive information bubble will also be added to improve the readability of icons.

##### Missing Elements

The interface displayed the recommendations and the elements required for antibiotic prescription according to the experts writing CPGs. However, the qualitative analysis of the free comments highlighted the following: (1) some properties were considered important by CPG experts but not by GPs (eg, CPG experts considered antibiotic taste to be important for improving treatment adherence, whereas this was not the case for all GPs); (2) GPs considered other properties not mentioned in CPGs to be important (eg, some GPs reported needing to visualize drug interactions, which are seldom dealt with in CPGs); and (3) some properties are currently used by CPGs experts but are not understood by GPs (eg, the property nonprecious class). This gap between real-life practice and writing CPGs should be considered when designing CDSS interfaces. Thus, interfaces should also include the properties used by GPs in real-life practice, even if the evidence for their use is poor or not given in CPGs. For example, with regard to antibiotic prescription, Krishnakumar et al [6] developed a model of the rationale used by GPs for antibiotic choice. Some of the factors included in this model, such as drug pharmacokinetics, marketing authorization, and drug cost, will be included in the web interface in the future.

#### Interface Evaluation

The participating GPs were recruited via emails sent to medical networks and by word of mouth. This mode of recruitment was chosen because it is faster and cheaper than traditional approaches [27,28]. However, it may have introduced a selective participation bias [28] (eg, by selecting GPs keen on the use of new technologies). This bias was limited by using large mailing lists (>850 GPs), including GPs working in various health care centers, from a large French region (including Paris). The readability of the interface and its utility for improving GPs’ comprehension of CPGs were also evaluated. Assessments of readability and comprehensibility are part of the software lifecycle and may prevent serious usability problems, which often occur during the design of new technologies [29]. Early testing makes it possible to identify serious issues and to improve the interface before its implementation in a CDSS.

### Comparison With Previous Work

#### Comparison With Other Drug Comparison Interfaces

Other types of interfaces have been developed for comparing drugs in medical domains. With regard to Dopamine [30], for instance, double-entry tables were used to compare the contraindications and side effects of drugs. More sophisticated tables, including the possibilities of overlapping data and interactions, have also been developed, such as Twinlist [31] for medication reconciliation or rainbow boxes [32,33] for comparing drug information such as contraindications or side effects. Rainbow boxes were previously adapted for use in antibiotic prescription [34]; the resulting rainbow interface was perceived as easy to read for 27.5%-64% of GPs, depending on the clinical situation. However, as smartphones are increasingly being used by medical doctors for learning, information retrieval, and/or clinical decision support [35-38], it is important to consider interfaces more suitable for use with a smartphone. Tabular representations take up a large amount of space and are therefore more suitable for computer interfaces [39,40]. Here, an e-commerce interface adaptable for smartphones was designed as a means of overcoming this limitation. *Graphs* have also been used for drug comparisons, such as Rxplore [41] for the rapid review of potential drug events caused by drugs and Network graph [42] for chemotherapy treatment. However, in these cases, graphical representations were interesting because of the need to add quantitative information such as the results of clinical trials [42]. As antibiotic properties are qualitative data, graphical representations were not considered appropriate for this situation.

#### Comparison With Other Antibiotic Prescription Interfaces

Other interfaces have been developed for the empiric prescription of antibiotics in primary care, for example, IAAP Smart phone [43], ARI Smart Form [44], and ABX TRIP [21] for acute respiratory infections. These interfaces display antibiotic recommendations without their justifications—that is, only the recommended antibiotics are displayed in a textual format without their properties. This type of presentation suffers from “the black box effect” [45] and may make GPs passive in their decision-making process, simply accepting the decision suggested by the CDSS. GPs may also be suspicious of the recommendations due to a lack of understanding of the suggestions made by the CDSS. In our proposed interface, drug properties were displayed to involve GPs in the decision process. This improves their understanding of the underlying reasons for recommendations and enables them to compare the advantages and disadvantages of the recommended antibiotics. Preserving GP autonomy is an important factor to be considered when trying to increase the chances of CDSS being successfully adopted [46].

### Conclusions

A web interface for antibiotic prescription presenting drug recommendations and their justifications was designed following the basic framework of an e-commerce interface. The justifications of CPG recommendations were displayed in the form of antibiotic properties, which was considered useful by more than three-fourths of GPs and helpful for understanding CPGs by two-thirds of GPs evaluating the interface. Further improvements are required before the implementation of this interface in a CDSS.

## Abbreviations

CPG: clinical practice guideline
CDSS: clinical decision support system
GP: general practitioner
VCM: Visualisation des Connaissances Médicales (Visualization of Medical Knowledge)
